# Determinants of Asthma Control in Jordanian Children: The Role of Comorbidities and FeNO Levels

**DOI:** 10.3390/children13010017

**Published:** 2025-12-22

**Authors:** Enas Al-Zayadneh, Walid Al-Qerem, Judith Eberhardt, Alaa Hammad, Ebaa Alzayadneh, Ahmad M. Elheet, Ruaa Allubani, Maryam A. Alani, Mina F. Al-Wandawi, Farooq Firas Al-Wandawi, Lama Adel Salem Rabadi, Joud Al Karmi, Montaha Al-Iede

**Affiliations:** 1 Department of Pediatrics, School of Medicine, University of Jordan, Queen Rania St., Amman 11942, Jordan; e.alzayadneh@ju.edu.jo (E.A.-Z.); m.al-iede@ju.edu.jo (M.A.-I.); 2 Department of Pharmacy, Faculty of Pharmacy, Al-Zaytoonah University of Jordan, Airport Street, Amman 11733, Jordan; alaa.hammad@zuj.edu.jo; 3 Department of Psychology, School of Social Sciences, Humanities and Law, Teesside University, Borough Road, Middlesbrough TS1 3BX, UK; j.eberhardt@tees.ac.uk; 4 Department of Physiology and Biochemistry, School of Medicine, University of Jordan, Queen Rania St., Amman 11942, Jordan; e.zayadneh@ju.edu.jo; 5 Blackpool Victoria Hospital, Whinney Heys Road, Blackpool, Lancashire FY3 8NR, UK; ahmad.elheet@nhs.net; 6 School of Medicine, The University of Jordan, Queen Rania St., Amman 11942, Jordan; rallubani@hcac.com.jo; 7 School of Graduate Studies, The University of Jordan, Queen Rania St., Amman 11942, Jordan; mry8240015@ju.edu.jo (M.A.A.); mina2102000@gmail.com (M.F.A.-W.); far0200016@ju.edu.jo (F.F.A.-W.); lma0194829@ju.edu.jo (L.A.S.R.); jod8231426@ju.edu.jo (J.A.K.)

**Keywords:** FeNO, asthma, spirometry, children, type two inflammation

## Abstract

Background/Objectives: Asthma is a prevalent chronic respiratory disease in children, and poor asthma control remains a major clinical challenge worldwide. In Jordan, the rising prevalence of pediatric asthma highlights a need to better understand the factors influencing asthma control and to evaluate new assessment tools. Methods: This cross-sectional study aimed to identify predictors of asthma control and to assess the clinical utility of fractional exhaled nitric oxide (FeNO) as a supplementary biomarker. A total of 329 children with physician-diagnosed asthma, aged 7–17 years, were recruited from Jordan University Hospital. Clinical history, spirometry, FeNO measurements, and Asthma Control Test scores were collected. Results: Overall, 62.6% of participants had uncontrolled asthma. Logistic regression analysis revealed that comorbidities such as obstructive sleep apnea, gastroesophageal reflux disease, allergic rhinitis, and bronchiolitis obliterans were significantly associated with poorer asthma control. Antihistamine use and elevated FeNO levels were also linked to lower odds of asthma control. Conclusions: While FeNO showed promise as a non-invasive marker of airway inflammation, its clinical application remains limited due to variability and confounding factors. A comprehensive, individualized approach to asthma management, considering comorbidities and conventional assessments, is essential. Larger, longitudinal studies are needed to establish the role of FeNO in routine pediatric asthma care.

## 1. Introduction

Asthma is a major global health concern, affecting nearly 300 million individuals worldwide and causing approximately 1000 deaths each day [1]. In recent years, the prevalence of asthma among children has been rising rapidly [2], with detection rates reaching 10%, making it a pressing global public health issue [3,4]. In Jordan, the prevalence of asthma among children and adolescents has increased significantly, from 4.3% in 1996 to 12.3% in 2011 [5,6]. Despite this significant increase, local data on the factors influencing asthma control, particularly in pediatric populations, remain limited, hampering the ability to formulate evidence-based management plans.

Asthma has long been defined as a heterogeneous syndrome characterized by airway hyperresponsiveness, which manifests clinically as cough, wheezing, and shortness of breath, with various triggers causing exacerbations and disease progression [7]. Despite the extensive body of research and proposed treatment modalities for pediatric asthma, uncontrolled disease continues to impose a significant physical, mental, and socioeconomic burden on both affected individuals and caregivers [8]. The rates for uncontrolled asthma were significantly high among U.S and Swedish children at 50.3% and 31.0%, respectively [9,10]. Such public health concern is best manifested as a significant economic burden at a global scale [11,12]. The U.S annual costs for asthma are estimated to be $82 billion [13].

Uncontrolled asthma in pediatrics exhibits a far-reaching impact beyond its challenging spectrum of treatment as it impacts academic performance and quality of life [14,15]. A Jordanian study showed that about 70% of children with asthma missed school due to their condition [16]. Another cross-sectional study evaluating asthma care in U.S. children aged 5 to 17 found that adolescents with poorer asthma-related emotional quality of life experienced worse asthma control and increased school absences [17]. Moreover, a retrospective cohort study of asthma patients treated at King Abdullah University Hospital found that severe, uncontrolled asthma was associated with significantly higher total annual medical costs [18].

There are many factors known to influence asthma control, such as comorbid conditions and medication adherence [19,20]. Associated comorbidities include are but are not limited to obstructive sleep apnea (OSA), gastroesophageal reflux disease (GERD), rhinitis, sinusitis, and bronchiolitis. These conditions can affect asthma through different pathophysiological mechanisms; for instance, OSA may contribute to asthma through systemic inflammation, while GERD can worsen asthma by directly irritating the airways [21]. A meta-analysis examining the relationship between various comorbid conditions and asthma found a strong correlation between allergic rhinitis and asthma, with approximately 30% to 80% of asthmatic patients also having allergic rhinitis [19,20].

Assessment of asthma control is essential for effective disease management and the prevention of exacerbations. Validated questionnaires (e.g., Childhood Asthma Control Test) [22], spirometry-based calculations (e.g., Global Lung Initiative equations) [23,24,25], or surrogate biomarkers (e.g., Exhaled breath condensate, urinary cysteinyl leukotriene E4, eosinophilic cationic protein) [26], were developed as means of assessing asthma control to facilitate timely intervention.

Recently, fractional exhaled nitric oxide (FeNO) has been proposed as a promising non-invasive tool for identifying asthma phenotypes, determining the level of asthma control, predicting exacerbations, and assessing the potential need for medication [27]. It is considered a reliable biomarker for detecting type 2 inflammation in asthma [28]; therefore, it is currently being studied for both asthma diagnosis and disease monitoring [29]. Numerous studies evaluating FeNO use in asthmatic children have found elevated FeNO levels during exacerbations, supporting its role in identifying airway inflammation [30]. The National Institute for Health and Care Excellence (NICE) recommends considering FeNO measurements to manage symptomatic asthma in patients who continue to experience symptoms despite the use of inhaled corticosteroids [31]. Moreover, the American Thoracic Society (ATS) has recommended FeNO testing since 2021 as a strategy to reduce exacerbations by tailoring asthma treatment [32].

In light of what’s above, the present cross-sectional study aimed to identify the key factors influencing asthma control status, including comorbidities, medication use, and reliance on rescue drugs. In addition, our study examined the association between FeNO levels and asthma control. By addressing these factors, we hoped to contribute to improving asthma control in pediatric patients, particularly within the Jordanian healthcare context where tailored data and national guidelines are still developing.

## 2. Materials and Methods

### 2.1. Study Design

This cross-sectional study included asthmatic children aged 7 to 17 years who were diagnosed according to the GINA criteria and were followed by pediatric pulmonary specialists. Diagnosis of asthma was established by expert physicians based on the presence of variable asthma-related symptoms (e.g., wheezes, shortness of breath, cough) and variable expiratory airflow limitation. Indicators of variability include significant bronchodilator reversibility, significant average daily diurnal PEF variability, or FEV_1_ increases of ≥12% after 4 weeks of anti-inflammatory treatment.

Children younger than 7 or older than 17 years, as well as those unable to perform FeNO or pulmonary function testing (PFT), were excluded. Clinical history was collected through a questionnaire completed by the children or their parents. All medical reporting was cross-checked with clinical records to reduce bias. The questionnaire covered a range of topics, including medication history, such as the use of inhaled corticosteroids, inhaled salbutamol, montelukast, antihistamines, and oral steroids. Additionally, family history of asthma and social factors were assessed, including exposure to environmental tobacco smoke, pet ownership, and the presence of eczema. OSA diagnosis was established through clinical examination and the Pediatric Sleep Questionnaire [33].

### 2.2. Study Population

The study was conducted at Jordan University Hospital (JUH) between August 2023 and April 2024. It involved 329 children with asthma who visited the respiratory clinics at JUH in Amman, Jordan. For each participant, clinical history, FeNO, PFT, and Asthma Control Test (ACT) scores were recorded. To ensure the stability and generalizability of the stepwise logistic regression model, the number of required sample size was based on the number of predictor variables as stated by the Events Per Variable (EPV) rule. As the final model included 6 predictors, the smallest group should include more than 60 children.

### 2.3. FeNo Device

Spirometry and FeNO assessments were conducted in accordance with established international guidelines to ensure accurate and reliable pulmonary function measurements. Spirometry was performed using a calibrated portable spirometer, strictly following the recommendations of the American Thoracic Society and the European Respiratory Society (ATS/ERS) for standardized lung function testing. Children were instructed to perform forced expiratory maneuvers after proper demonstration and practice. Multiple attempts were allowed to obtain at least three acceptable and reproducible curves, from which the highest values of FEV_1_ and FVC were recorded.

FeNO testing was conducted using the NObreath^®^ device (Kent, United Kingdom), in line with ATS guidelines for clinical FeNO measurement. Prior to testing, children were asked to refrain from eating, drinking, or engaging in strenuous physical activity for at least one hour. The test required a steady exhalation at a constant flow rate and was repeated if the exhalation did not meet the required criteria. All assessments were conducted under the supervision of trained personnel to ensure proper technique and adherence to procedural protocols.

### 2.4. Asthma Control

The severity of asthma was evaluated based on medical history prior to FeNO measurement. The ACT was used to assess asthma control. It includes five questions that evaluate the frequency of shortness of breath, nighttime asthma symptoms, functional limitations, use of rescue medications, and the patient’s self-assessment of their asthma control. Each question offers five response options, scored from 1 to 5. Based on the total score, asthma control was categorized as follows: well-controlled (20–25), partially controlled (15–19), and uncontrolled (5–14). Additionally, we used the GINA criteria, which assess asthma severity based on four questions regarding symptoms over the past four weeks. These include: (1) having daytime symptoms more than twice per week, (2) any nighttime waking due to asthma, (3) use of a short-acting beta-agonist (SABA) more than twice per week, and (4) any activity limitation due to asthma.

For analysis purposes, each “yes” response was scored as 1 point and each “no” as 0. Scores were then classified as: well-controlled (0 points), partly controlled (1–2 points), and uncontrolled (3–4 points).

### 2.5. Ethical Considerations

The study was conducted in compliance with the Declaration of Helsinki. The study protocol and proposal were reviewed and approved by the Institutional Review Board (IRB) of Jordan University Hospital (IRB #10562-2023, 11 July 2023). Written informed consent was obtained from the parents or legal guardians of all study participants.

### 2.6. Statistical Analysis

Data analysis was conducted using SPSS version 26 IBM Corp., Armonk, NY, USA. Descriptive statistics were reported as medians and interquartile ranges (25–75 percentiles) for continuous variables, while categorical variables were summarized using frequencies and percentages.

Bivariate analysis was performed to assess the association between various independent variables and asthma control status. The chi-square test was used for categorical variables, the Mann–Whitney U test for non-parametric continuous variables, and the *t*-test for normally distributed continuous variables.

A forward stepwise binary logistic regression model was used to identify factors significantly associated with asthma control, as measured by the ACT. Variables with a *p*-value ≤ 0.20 in the bivariate analysis were included as predictors in the model. A *p*-value of < 0.05 was considered statistically significant.

## 3. Results

### 3.1. Sociodemographic Characteristics

Table 1 presents the sociodemographic characteristics of the 329 children with asthma included in the study. Fifty-nine percent were male, and the median age was 11 years (interquartile range: 9–13 years). Most children were born at term (91.2%), and 50.5% had a healthy weight status. More than half of the participants had a family history of asthma (56.8%) and had been previously hospitalized due to asthma (59%). Additionally, 74.5% of the children were diagnosed with obstructive sleep apnea (OSA), and the median duration since asthma diagnosis was 12 months (range: 2–48 months).

### 3.2. Comorbidities and Medication Use

Table 2 summarizes the comorbidities and medications reported by study participants. The majority had allergic rhinitis (67.2%), and 53% had suppurative lung disease (wet cough). Most children were using antihistamines (71.1%), and 50.5% had received oral steroids in the past year. Additionally, 57.1% of participants used short-acting beta-agonist (SABA) inhalers. About 26% of SABA users reported needing them 1 to 2 times per day during the last 7 days. Additionally, 45.9% of included participants used Fluticasone Propionate (i.e., Flixotide), which was the most commonly used inhaled corticosteroid/long-acting beta-agonist (ICS/LABA) medication.

Table 3 presents the results of the medical tests conducted in this study. Based on ACT scores, 62.2% of children had uncontrolled asthma, while 42.6% had a positive skin prick test. The median FEV_1_% was 89.82 (interquartile range: 77.92–100.95), and the median FEV_1_/FVC ratio was 84% (77.2–91.1). The median FeNO level was 14 parts per billion (ppb), with a range of 9–26 ppb. Per ATS guidelines, the included cohort was distributed as follows: 62.9% (n = 207, FeNO < 20 [Low]), 19.5% (n = 64, 20 < FeNO < 35 [Middle]), and 17.6% (n = 58, FeNO > 35 [High]).

### 3.3. Factors Associated with Asthma Control

A stepwise binary logistic regression model was used to identify significant predictors of asthma control status (Table 4). Children without OSA had significantly higher odds of being in the controlled asthma group compared to those with OSA (OR = 4.825, 95% CI: 2.648–8.790, *p* < 0.001). Similarly, children without GERD (OR = 7.275, 95% CI: 1.277–41.437, *p* = 0.025), allergic rhinitis (OR = 1.750, 95% CI: 1.006–3.046, *p* = 0.048), or bronchiolitis obliterans (OR = 3.577, 95% CI: 1.947–6.573, *p* < 0.001) also had significantly greater odds of asthma control.

In addition, children who did not use antihistamines had increased odds of being in the controlled group (OR = 2.021, 95% CI: 1.142–3.575, *p* = 0.016). Finally, higher FeNO levels were associated with lower odds of asthma control (OR = 0.983, 95% CI: 0.970–0.997, *p* = 0.014).

## 4. Discussion

This study investigated asthma control and its determinants among children attending respiratory clinics at Jordan University Hospital. A high proportion (62.6%) had uncontrolled asthma, emphasizing the clinical burden and the importance of understanding contributing factors, as the main goal of asthma care is to achieve and maintain control [34]. The rate of uncontrolled asthma aligns with findings from Saudi Arabia (59–84%) [35], the US (46%) [36], and Southeast Nigeria (64.4%) [37], but contrasts with lower rates reported in other settings; 18% in another Nigerian centre [38], 31% in Ethiopia [39], and 40% in a multiethnic Dutch sample [40]. These differences may reflect variations in healthcare access, parental awareness, environmental exposures, asthma severity, and the tools used to assess control [40,41,42,43]. Consistent with previous studies, bronchial asthma was more prevalent in males under 14 years [44]; in our sample, 59% were boys. Although the reasons remain uncertain, genetic, environmental, and behavioural factors are likely involved.

As in prior research, comorbid conditions were strongly associated with poorer asthma control. OSA, present in 74.5% of our sample, significantly reduced the likelihood of asthma control. This differs from findings in a small US study of non-obese African American children (1.9% OSA prevalence), which reported no association [45]. GERD was also linked to poor control, likely due to reflux-related airway inflammation [46].

Allergic rhinitis, found in 67% of participants, is a well-established asthma comorbidity. It has been shown to worsen asthma symptoms, particularly when untreated [47]. In our study, its presence was significantly associated with uncontrolled asthma. This is consistent with research by Adams et al. [48], who found that over 22% of children with asthma also had both allergic rhinitis and eczema.

Bronchiolitis obliterans, identified in one-third of participants, was another significant factor. Prior studies have linked bronchiolitis in early childhood to subsequent asthma development and poor control [49,50]. Our findings support this association and highlight the long-term implications of early respiratory illness. Multiple reports indicate the overlapping clinical, functional, radiological, and atopic characteristics between bronchiolitis obliterans and asthma, which may lead to misdiagnosis and worse treatment outcomes [51,52]. In our cohort, both disease entities coexisted and were associated with poor asthma control. We believe that such relationship stems from similar pathological mechanisms, particularly those related to Th17 immune function, Regulatory T cells, and neutrophil dysfunction; all of which are commonly observed in both disease entities [53]. Similarly, pulmonary immune profiling demonstrated a link between suppurative lung disease and childhood wheezing [54]. Ruffles et al., demonstrated that among children affected with protracted bacterial bronchitis, the mildest manifestation of suppurative lung disease, about 25% developed asthma with two thirds demonstrated bronchodilator reversibility [55]. Immune phenomenon linking both disease entities include neutrophilic infiltration, elevated signatures of the IL-4/IL-13 pathways, and Th2 dysregulation [56].

Antihistamine use was associated with poorer asthma control in our sample. While antihistamines may improve outcomes in children with allergic asthma [57], they may be less effective, or even harmful, in those with non-allergic phenotypes, potentially increasing airway constriction and inflammation [58]. Nonetheless, the association between antihistamines and asthma in our cohort could be attributed to the confounding by indication bias due to the high rates of allergic rhinitis, which is also associated with antihistamine usage. Interestingly, the predictive value of antihistamines remained significant across all steps of the multivariate model; thus, indicating some form of statistical independence. As per the 2022 GINA guidelines, antihistamines are not considered as a viable management option for asthma and is only reserved for when it co-exists with other allergic comorbidities [59].

FeNO levels were another key factor. Children with elevated FeNO had significantly lower odds of asthma control. FeNO is a sensitive marker of eosinophilic inflammation and has been shown to predict exacerbations. Our findings are consistent with a Thai study, where children with higher FeNO and a history of exacerbations were more likely to experience future episodes [60].

Within the Jordanian healthcare context, FeNO could serve as a cost-effective non-invasive adjuvant diagnostic and monitoring measure for pediatric patients with asthma. While FeNO adds value to asthma assessment, its interpretation can be influenced by atopy, infections, and other variables [28,29]. Therefore, FeNO should complement—not replace—symptom-based tools such as the ACT and spirometry. Moreover, cut-off values should be psychometrically validated against a Jordanian normative data as current FeNO thresholds significant vary between guidelines (e.g., NICE, GINA, EPR-4) and are mostly influenced by the characteristics of the cohorts on which they were calculated [29].

The utility of FeNO is bound to a number of other technical and clinical challenges. Kim et al., demonstrated in their review that FeNO is sensitive to a number of factors including age, gender, race, smoking, and environmental pollution [61]. Moreover, changes in FeNO are not specific to a single atopic condition, let alone all phenotypes of asthma [62]. On the other hand, while the measurement of FeNO has standardized guidelines, younger pediatric patients (i.e., <5 years old) may not be as compliant.

Nevertheless, FeNO could be utilized in the diagnosis pathway of steroid-naïve pediatrics, of whom symptoms and signs after inconclusive after standard spirometry or bronchodilator reversibility testing [1]. However, the GINA 2024 report still considers FeNO as an inconclusive tool in terms of its diagnostic prowess [27]. Moreover, FeNO could be used as a measure of adherence to ICS therapy or during step-down decisions from ICS therapy [27]. FeNO could also be used to assess phenotypic eligibility and predict response for a number of biologic agents in cases of severe asthma [63].

In summary, this study reinforces the complexity of asthma control in children and the multifactorial nature of poor control. Comorbidities including OSA, GERD, allergic rhinitis, and bronchiolitis obliterans, as well as medication use (e.g., antihistamines) and elevated FeNO levels, were all associated with poorer outcomes. Our findings point to the importance of a tailored, multidisciplinary approach to asthma management in pediatric populations, especially in settings like Jordan, where local data have been limited.

### Strengths, Limitations, and Future Directions

This study is among the first in Jordan to examine asthma control in children using both clinical assessments and validated symptom-based tools. Its strengths include a relatively large sample and detailed evaluation of comorbidities and medication use, which enhance the reliability of findings.

However, the cross-sectional design limits causal inference. We also lacked detailed data on medication adherence and duration, which may have influenced outcomes. Moreover, it hampered our ability to investigate the relationship between FeNO levels and level of adherence. FeNO levels can be affected by allergic sensitization, previous medications, infections, environmental factors, and demographic differences; all of which were not fully accounted for. Asthma control was established by the ACT tool. Despite its reliability, it is a subjective measure of control that could be influenced by parental bias or recall bias of older participants. Finally, as the study was conducted in a single tertiary centre, generalisability to broader pediatric populations, including those in primary care or rural settings, is limited.

Future research should use longitudinal, multicentre designs with more diverse samples to clarify causal relationships and improve external validity. Studies should also monitor medication use more closely and explore the biological pathways linking comorbidities such as OSA, GERD, and allergic rhinitis to asthma control.

## 5. Conclusions

This study found that higher FeNO levels were significantly associated with poorer asthma control in children, supporting its potential as a supplementary biomarker of airway inflammation. However, its clinical use remains limited due to variability and the influence of confounding factors.

Comorbid conditions such as OSA, GERD, allergic rhinitis, and bronchiolitis obliterans were also strongly linked to uncontrolled asthma. These findings highlight the importance of a comprehensive and multidisciplinary approach to asthma management. The association between antihistamine use and poor asthma control further suggests that treatment should be tailored to individual clinical profiles.

FeNO may be useful in identifying eosinophilic inflammation and guiding targeted therapy, but it should be used alongside, rather than instead of, established tools such as the ACT, GINA criteria, and spirometry. Its integration into routine care in Jordan could be beneficial if applied within a broader clinical context.

Overall, effective pediatric asthma management requires an individualized strategy that incorporates symptom assessment, clinical history, pulmonary function testing, and careful evaluation of comorbidities. Larger, longitudinal studies are needed to validate FeNO thresholds and clarify its predictive value for long-term outcomes.

## Figures and Tables

**Table 1:** Sociodemographic characteristics of the sample.

		Count (%) or Median (25–75) Percentiles
Age		11 (9–13)
Gender	Female	135 (41%)
	Male	194 (59%)
Birth weight (kg)		3 (2.60–3.50)
Weight status category	Underweight	22 (6.7%)
	Healthy Weight	166 (50.5%)
	Overweight	64 (19.5%)
	Obesity	77 (23.4%)
Gestational age	Preterm	29 (8.8%)
	Term	299 (91.2%)
Diagnosis duration in months		24 (0–72)
Previous admission due to asthma		194 (59%)
Family history of asthma		187 (56.8%)
Smoking Exposure		173 (52.6%)
Pet Exposure		127 (38.6%)
Obstructive sleep apnea (OSA)		245 (74.5%)

**Table 2:** Comorbidities and medication use among study participants.

		Count (%)
Eczema/Atopic dermatitis		53 (16.1%)
Allergic rhinitis		221 (67.2%)
Food allergy		37 (11.3%)
Penicillin allergy		17 (5.2%)
Cystic fibrosis		7 (2.1%)
Chronic lung disease of prematurity		3 (0.9%)
Bronchiolitis obliterans		111 (33.8%)
Suppurative lung disease (wet cough)		174 (53%)
GERD		16 (4.9%)
LABA		88 (26.8%)
Type of ICS/LABA medication used	Clenil (Beclometasone)	13 (7%)
	Flixotide (Fluticasone Propionate)	85 (45.9%)
	Foster (Beclometasone/Formoterol)	1 (0.5%)
	Seretide (Salmeterol/Fluticasone Propionate)	68 (36.8%)
	Symbicort (Budesonide/Formoterol)	18 (9.7%)
SABA		188 (57.1%)
Duration of SABA use (months)		12 (2–48)
Frequency of SABA inhaler use in the past week	Not at all	51 (27.1%)
	Once a week	28 (14.9%)
	More than 3 times per week	21 (11.2%)
	1 or 2 times per day	49 (26.1%)
	3 or more times per day	39 (20.7%)
Montelukast		35 (10.6%)
Antihistamine		234 (71.1%)
Use of oral steroids in the past year		166 (50.5%)

**Table 3:** Results of medical tests among study participants.

		Median (25–75) Percentiles or Count (%)
Fractional exhaled nitric oxide (PPB)		14 (9–26)
FVC (L)		2.32 (1.83–3.01)
FEV_1_ (L)		1.9 (1.5–2.53)
FEV_1_/FVC%		84 (77.2–91.1)
FEV_1_%		89.82 (77.92–100.95)
Asthma Control Test (ACT) classification	Uncontrolled asthma	206 (62.6%)
	Controlled asthma	123 (37.4%)
Skin prick test	Not available	112 (34%)
	Positive	140 (42.6%)
	Negative	77 (23.4%)

**Table 4:** Binary regression of the variables associated with participants’ asthma control (Uncontrolled vs. Controlled).

		OR	*p*-Value	95% CI for OR	
				Lower	Upper
Step 1	* OSA	5.365	<0.001	3.111	9.253
	Constant	0.373	<0.001		
Step 2	* OSA	4.732	<0.001	2.699	8.294
	* Bronchiolitis obliterans	3.313	<0.001	1.849	5.936
	Constant	0.166	<0.001		
Step 3	* OSA	4.607	<0.001	2.616	8.115
	* Bronchiolitis obliterans	3.293	<0.001	1.823	5.948
	* Antihistamine	2.029	0.011	1.179	3.492
	Constant	0.134	<0.001		
Step 4	* OSA	4.737	<0.001	2.663	8.426
	* Bronchiolitis obliterans	3.547	<0.001	1.950	6.451
	* Antihistamine	2.183	0.006	1.253	3.804
	Fractional exhaled nitric oxide	0.984	0.014	0.971	0.997
	Constant	0.175	<0.001		
Step 5	* OSA	5.208	<0.001	2.876	9.433
	* GERD	6.829	0.027	1.249	37.334
	* Bronchiolitis obliterans	3.651	<0.001	1.992	6.691
	* Antihistamine	2.198	0.006	1.252	3.856
	Fractional exhaled nitric oxide	0.982	0.008	0.969	0.995
	Constant	0.027	<0.001		
Step 6	* OSA	4.825	<0.001	2.648	8.790
	* Allergic rhinitis	1.750	0.048	1.006	3.046
	* GERD	7.275	0.025	1.277	41.437
	* Bronchiolitis obliterans	3.577	<0.001	1.947	6.573
	* Antihistamine	2.021	0.016	1.142	3.575
	Fractional exhaled nitric oxide	0.983	0.014	0.970	0.997
	Constant	0.021	<0.001		

* No versus Yes.

## Data Availability

The dataset supporting the conclusions of this article is available in the Zenodo repository, https://doi.org/10.5281/zenodo.15294818.
